# Exploring the association between early adaptive schemas and self‐reported eating disorder symptomatology

**DOI:** 10.1002/cpp.2789

**Published:** 2022-10-17

**Authors:** Anthea L. Maher, Andrew Allen, Jonathan Mason, Catherine Houlihan, Andrew P. Wood, Tyrone Huckstepp

**Affiliations:** ^1^ School of Health and Behavioural Sciences University of the Sunshine Coast Sippy Downs Queensland Australia; ^2^ Sunshine Coast Mind and Neuroscience—Thompson Institute University of the Sunshine Coast Birtinya Queensland Australia; ^3^ Eating Disorder Service Queensland Hospital and Health Service Maroochydore Queensland Australia

**Keywords:** early adaptive schema, early maladaptive schema, eating disorder, schema therapy

## Abstract

**Objective:**

The current study aimed to examine the relationship between early adaptive schemas and eating disorder symptomatology in adults.

**Method:**

A cross‐sectional, correlational design was used to collect data from 352 females and 36 males aged between 18 and 49 years (*M* = 25.70, *SD* = 7.04). Participants completed an online questionnaire, which included The Young Positive Schema Questionnaire (YPSQ), Eating Disorder Examination‐Questionnaire (EDE‐Q) and demographic measures.

**Results:**

Four separate hierarchical multiple regression analyses showed that high levels of Healthy Boundaries and low levels of Optimism significantly predicted lower Restraint, Eating Concern, Shape Concern and Weight Concern scores. Additionally, higher scores in Emotional Openness and Social Belonging significantly predicted lower Eating Concern, while higher scores in Self‐Care significantly predicted lower levels of Shape Concern.

**Conclusion:**

The findings highlight the protective function that certain early adaptive schemas may play in mitigating eating disorder symptomatology. Moreover, the findings allude to potential modifiable therapy targets in the treatment of eating disorders. Further research is needed to investigate any differences in early adaptive schemas between eating disorder diagnoses.

**Key Practitioner Message**

- Higher Optimism scores predicted worse Restraint, Shape Concern, Weight Concern and Eating Concern scores
- Higher scores in Healthy Boundaries, Emotional Openness, Social Belonging and Self‐Care predicted lower scores in a variety of EDE‐Q outcome measures, including Restraint, Shape Concern, Weight Concern and Eating Concern
- A unique set of early adaptive schemas may mitigate the development of pathological eating cognitions and behaviours

## 1 INTRODUCTION

Eating disorders are accompanied by some of the highest fatality rates across mental disorders (Chesney et al., 2014). According to systematically reviewed data, lifetime estimates of eating disorders range from 8.4% for women and 2.2% for men, with a combined global increase in prevalence of 4.3% over the past 15 years (Galmiche et al., 2019). Given the serious consequences associated with eating disorders, encompassing physical (e.g., malnourishment, heart failure and cerebral atrophy) and psychological complications (e.g., suicide, depression and reduced quality of life; e.g., Arcelus et al., 2011; Smith et al., 2019; Winkler et al., 2014), further research is required to improve prognostic and treatment outcomes. Eating disorders are characterized by negative values and attitudes concerning body weight and shape. Moreover, individuals with eating disorders tend to judge themselves almost exclusively in terms of their eating, shape or weight (Fairburn et al., 2003). Across diagnostic categories, presentations comprise either exclusively or a combination of caloric restriction (e.g., anorexia nervosa), binge eating (e.g., binge eating disorder) or purging behaviour (e.g., bulimia nervosa). Although, more than half of individuals receiving treatment fall within the diagnostic category of eating disorder not otherwise specified (OSFED), which manifests as characteristic symptoms that do not meet the criteria of a specific eating disorder diagnostic category (American Psychiatric Association, 2013).

The most widely researched and accepted theoretical framework for the treatment of eating disorders is cognitive behavioural therapy (CBT). Early models of CBT emphasized the roles in which disordered eating and maladaptive attitudes towards weight and shape contributed to the maintenance of eating disorder pathology (Fairburn, 1981). Building on this premise, Fairburn and colleagues (Fairburn et al., 2003) subsequently proposed a transdiagnostic model and ‘enhanced’ form of CBT (CBT‐E), detailing four additional features of eating disorder maintenance: (1) low self‐esteem, (2) perfectionism, (3) interpersonal difficulties and (4) mood intolerance. The transdiagnostic model suggests that all eating disorder diagnoses display shared but distinctive, clinical features that tend to be maintained by similar psychopathological processes. A recent systematic review of 20 studies provided support for CBT‐E as an effective treatment for adults with a range of eating disorder diagnoses (Atwood & Friedman, 2020). de Jong and colleagues, using a randomized controlled trial, also reported significant effectiveness in the use of CBT‐E compared with usual treatment (de Jong et al., 2020).

Despite the noted efficacy of CBT and its proponents (i.e., CBT‐E) for some eating disorder presentations such as bulimia nervosa and binge eating disorder, long‐term treatment outcomes have remained inconsistent (e.g., Agras et al., 2000; Poulsen et al., 2014; Waller et al., 2014). Specifically, literature (i.e., Atwood & Friedman, 2020; de Jong et al., 2020) has noted that eating disorder psychopathology improved in the short term but was not maintained, compounded by significant participant attrition. Several factors have been implicated in limited treatment outcomes for certain eating disorder populations such as entrenched cognitive schemata, problematic attachment styles and developmental trauma, treatment drop‐out and complex comorbidities (e.g., personality disorders; Vall & Wade, 2015). Such findings suggest that the current CBT model is necessary but not sufficient to conceptualize eating disorders, and further research is required to clarify and extend existing conceptualizations, which may contribute to improved outcomes in the field (Klump et al., 2009).

Given the aforementioned complexities associated with treatment, schema theory (Young, 1990; Young et al., 2003) may provide a complementary framework for explaining the aetiological and maintaining factors associated with eating disorders. Schema therapy, originally developed to expand on traditional CBT, endeavours to address developmental processes that contribute to and maintain psychopathology such as temperament, attachment and adverse early childhood experiences (Young, 1990; Young et al., 2003). The overlap of schema therapy with eating disorder pathology derives from evidence that highlights that individuals with eating disorders endorse both disorder‐specific cognitions and unconditional negative beliefs about the self, others and the world (Hughes et al., 2006). Such evidence aligns with Young and colleagues (Young et al., 2003) description of early maladaptive schemas, which are a central tenet of schema therapy. Defined as pervasive themes regarding the self, early maladaptive schemas are thought to develop in response to consistently unmet emotional needs during childhood and contribute to the aetiology and maintenance of psychopathology (Young et al., 2003). There are currently 21 proposed early maladaptive schemas, with the inclusion of three recent additions (Arntz et al., 2021), which fall under five domains and are outlined in Table 1. Thus, the schema model, developed from a range of psychological theories, aims to treat early maladaptive schemas through the alteration of entrenched negative life patterns, that is, cognitive and behavioural changes (Young et al., 2003).

**TABLE 1:** Overview of early maladaptive schemas and their theoretical counterpart early adaptive schema subsumed in schema domains and associated needs

Domain	Early maladaptive schema	Early adaptive schema^a^	Associated needs
Disconnection and rejection	Emotional deprivation	**Emotional fulfilment**	Secure attachment Acceptance Nurturing Protection
	Abandonment	**Stable attachment**	
	Mistrust/abuse	Basic trust	
	Social isolation/alienation	**Social belonging**	
	Defectiveness, shame	Self‐acceptance, lovability	
Impaired autonomy, competence and identity	Failure to achieve	**Success**	Autonomy Competence Identity
	Dependence, incompetence	**Self‐reliance/competence**	
	Vulnerability to harm	**Basic health and safety, optimism**	
	Enmeshment, undeveloped self	**Healthy boundaries, developed self**	
Impaired limits	Entitlement, grandiosity	**Empathic consideration**	Realistic limits Self‐control
	Insufficient self‐control	**Healthy self‐control, self‐discipline**	
Other‐directedness	Subjugation	Assertiveness, self‐expression	Free expression of needs and emotions
	Self‐sacrifice	**Healthy self‐interest, self‐care**	
	Approval‐seeking	**Self‐directedness**	
Exaggerated vigilance, inhibition	Emotional inhibition	**Emotional openness/spontaneity**	Spontaneity and play
	Unrelenting standards	**Realistic expectations**	
	Negativity, pessimism	Optimism, hopefulness	
	Punitiveness	**Forgiveness, self‐compassion**	
Recent additions	Lack of coherent identity Lack of meaningful world	n/a (Arntz et al., 2021)	Self‐coherence
Recent addition	Lack of fairness	n/a (Arntz et al., 2021)	Fairness

^a^ Theoretical counterparts of early maladaptive schemas derived from Louis et al. (2018). Note that early adaptive schemas in bold were the final constructs, as outlined in Louis et al. (2018).

Over two decades of research has documented the relationship between early maladaptive schemas and eating disorder symptomatology. Studies using clinical samples have found that individuals, regardless of eating disorder diagnosis, consistently report significantly higher early maladaptive schema scores than their nonclinical counterparts (e.g., De Paoli et al., 2017; Jones et al., 2005; Legenbauer et al., 2018; Leung et al., 2000; Leung & Price, 2007; Maher et al., 2022; Sines et al., 2008). Moreover, positive associations between early maladaptive schemas and a range of eating disorder measures such as drive for thinness, body dissatisfaction, bulimic symptoms and binge eating have emerged in nonclinical samples (e.g., Hovrud et al., 2020). Building on this evidence, Waller and colleagues (Waller et al., 2007) developed a schema‐focussed model, which proposed that eating disorders are used as an overcompensatory mechanism to avoid intense affect associated with early maladaptive schema activation, that is, exposure to individuals or situations that resemble previously unmet emotional needs. That is, intense emotions are avoided either prior to (e.g., caloric restriction, compulsive exercise) or after (e.g., binge eating and purging) schema activation, which functions to reinforce early maladaptive schema content (Waller et al., 2007). Taken together, the model indicates that while eating disorder diagnoses can be differentiated by behaviours and schema processes, schema content is transdiagnostically consistent across eating disorder symptomatology.

Since its conception, the schema model has been used to inform eating disorder treatment. Overall, modification of schema content using group programs has been successful in reducing the severity of schemas and eating disorder pathology (e.g., Mącik & Sas, 2015; Simpson et al., 2010). Such findings suggest that schema content is amenable to change in the therapeutic setting. However, given the complex and pervasive nature of eating disorder pathology, improvement of therapeutic outcomes remains an ongoing focus of research. Seligman, a proponent of positive psychology, proposed that increasing the focus on positive psychological outcomes and interventions may strengthen traditional interventions that tend to emphasize the reduction of psychopathology. In a 2005 article examining the progress of the field, Seligman et al. (2005) found moderate to large effect sizes for clinical interventions aiming to increase happiness. More importantly, a reduction in depressive symptoms was noted and happiness scores were maintained 6 months after the cessation of treatment, illustrating the possible effectiveness of positive psychology interventions. Recently, the field of positive clinical psychology looked at the unique contribution of positive psychological constructs in predicting mental health outcomes (Wood & Joseph, 2010). Moreover, the field has drawn attention to research that found that psychological interventions focussed on increasing positive psychological constructs have been as successful in reducing psychopathology as those that focus on reducing negative constructs (Geraghty et al., 2010). For example, a recent meta‐analysis (Hendriks et al., 2020) and systematic review (Chakhssi et al., 2018) found that increasing positive emotions, cognitions and behaviours resulted in improvements in depression, anxiety and stress scores for both clinical and nonclinical populations. Such findings allude to the notion that positively framed measures and treatment targets may have an important role to play in eating disorder treatment.

Given the burgeoning field of positive psychology, Louis and colleagues (Louis et al., 2018) recently developed a measure of positive schemas, with 14 theoretical counterparts to early maladaptive schemas, as outlined in Table 1. Named early adaptive schemas, they consist of positive functions and adaptive behavioural dispositions that emerge during childhood and adolescence when one's core emotional needs are adequately met (Young et al., 2003). Preliminary research found a significant negative relationship between early adaptive schema scores and depression, anxiety and stress (Louis et al., 2018). That is, individuals with positive core beliefs regarding themselves, others and the world were more likely to experience positive mental health outcomes. Within the eating disorder field, a dearth of research has examined the relationship between positive psychological constructs and eating disorder symptomatology. One study has examined the association between positive core beliefs and eating disorder symptoms in a nonclinical sample, which found that individuals who endorsed positive individual self‐beliefs (e.g., I am a capable person; I am resilient; I am independent) were more likely to endorse lower levels of self‐reported eating disorder symptoms (Cooper & Proudfoot, 2013). Such findings suggest that development of positive beliefs about oneself and the world may be protective against eating disorder cognitions and behaviours. Therefore, examining the nature of the relationship between positive psychological constructs such as early adaptive schemas and eating disorder symptomatology may have clinical value, given the lack of such measures and positive therapeutic targets in the eating disorder field.

## 2 PRESENT STUDY

Despite the prevalence of eating disorders having increased over the past two decades, treatment outcomes remain inconsistent (e.g., Agras et al., 2000; Poulsen et al., 2014; Waller et al., 2014). Considering the complex nature of eating disorders, schema theory may augment the current CBT‐based conceptualization, which is supported by findings that individuals with eating disorders report significantly higher early maladaptive schema scores than their nonclinical counterparts (e.g., De Paoli et al., 2017; Legenbauer et al., 2018; Leung & Price, 2007; Maher et al., 2022; Sines et al., 2008). Additionally, positive individual and social self‐beliefs have been negatively correlated with eating disorder symptoms in nonclinical women (Cooper & Proudfoot, 2013), suggesting that positive psychological constructs may be predictive of lower eating disorder symptomatology. Early adaptive schemas are a promising avenue of research and have been associated with positive mental health outcomes in preliminary studies (Louis et al., 2018). Given the paucity of literature examining positive beliefs and eating disorder symptomatology, research is required to elucidate the nature of this relationship. Examination of these variables is an important step in clarifying a schema‐based conceptualization of eating disorders and identifying potential positive psychological treatment targets for this population. Considering the dearth of research, the scope of the present study was exploratory in nature. Therefore, the aim of this study was to examine which, if any, early adaptive schemas predicted eating disorder symptomatology outcomes in adults.

## 3 METHOD

### 3.1 Participants

Participants were 36 males (9.3%) and 352 females (90.7%) recruited from social media. Age ranged from 18 to 49 years (*M* = 25.7, *SD* = 7.04) and body mass index (BMI) ranged from underweight (11.94 kg/m^2^) to obese (43.83 kg/m^2^; *M* = 19.76, *SD* = 5.59). More than two‐thirds of participants had completed vocational education or training such as a diploma (14.7%), bachelor degree (40.2%), master's degree (14.2%) or PhD (2.3%), while a quarter of participants had completed secondary school (25.8%) and 2.8% had completed Year 10 as their highest level of education. Most participants identified as North American (47.7%), while other participants identified as Australian (14.4%), British (11.2%), European (7.4%) or other nationality (6.3%). Just over half of participants (50.8%) reported having a current eating disorder diagnosis from a registered health professional, although only 14.4% were receiving treatment for their diagnosis, while nearly a quarter (24.2%) had previously received treatment and more than half (57.7%) had never received treatment.

### 3.2 Design

The study was a correlational, cross‐sectional online survey. Sixteen predictor variables and four outcome variables were included in the study. Predictor variables included age, BMI, and early adaptive schemas. Eating disorder symptomatology was the outcome variable.

### 3.3 Materials

#### 3.3.1 Demographic measures

Demographic information included sex, age, nationality, height, weight, education level and current mental disorder diagnosis.

#### 3.3.2 Early adaptive schemas

The Young Positive Schema Questionnaire (YPSQ; Louis et al., 2018) is a 56‐item scale designed to measure positive thinking patterns and experiences (i.e., positive schemas) and includes 14 subscales. Items are rated on a 6‐point Likert scale that ranges from a score of 1 (*Completely untrue of me*) to a score of 6 (*Describes me perfectly*). Sample items include the following: “I like to do well but don't have to be the best” (Realistic Expectations) and “I trust that people won't leave me, do I don't act needy and drive them away” (Stable Attachment). Scores are averaged for the total scale and each subscale, with higher scores reflecting greater endorsement of positive schemas. The YPSQ has demonstrated moderate internal consistency in nonclinical samples, with Cronbach's alpha ranging from .62 to .93 (Louis et al., 2018). The 14 subscales (Emotional Fulfilment, Success, Empathic Consideration, Optimism, Emotional Openness, Self‐Compassion, Healthy Boundaries, Social Belonging, Healthy Self‐Control, Realistic Expectations, Self‐Directedness, Self‐Care, Stable Attachment and Self‐Reliance) are negatively correlated with their early maladaptive schema counterpart, demonstrating construct validity. Additionally, correlations between the 14 subscales and measures such as depression, anxiety and life satisfaction provide evidence for convergent validity. Cronbach's alpha for the 14 subscales in the current sample ranged from .75 to .93.

#### 3.3.3 Eating disorder symptomatology

The Eating Disorder Examination Questionnaire (EDE‐Q; Fairburn & Beglin, 1994) is a 28‐item self‐report version of the interview‐based Eating Disorders Examination (EDE; Fairburn & Cooper, 1993). The EDE‐Q measures four aspects of eating disorder pathology, focusing on the previous 4 weeks; Restraint, Eating Concern, Shape Concern and Weight Concern. Items are scored on a 7‐point scale ranging from 0 (*No days*) to 6 *(Every day*). Sample items include the following: “Have you been deliberately trying to limit the amount of food you eat to influence your shape or weight (whether or not you have succeeded)?” (Restraint) and “Have you had a definite fear of losing control over eating?” (Eating Concern). Scores are averaged to obtain four subscale scores and a global score, with higher scores indicating more severe eating disorder pathology. The measure also addresses key behavioural aspects of eating disorders, including reported frequency of objective bingeing and purging episodes over a 28‐day period, although these items were not reported in the present study. The EDE‐Q has shown moderate internal consistency in women with various eating disorder diagnoses; Cronbach's alpha ranged from .58 to .78 for the Restraint subscale, .44 to .78 for the Eating Concern subscale, .68 to .85 for the Shape Concern subscale and .51 to .76 for the Weight Concern subscale (Berg et al., 2012). The EDE‐Q has also demonstrated temporal stability over a 2‐week period, with test–retest reliability coefficients ranging from .66 to .94 for scores on the four subscales (Berg et al., 2012). Cronbach's alpha for the four subscales in the current sample ranged from .84 to .93.

### 3.4 Procedure

After obtaining institutional ethical approval, participants were recruited via social networking websites with a link to the online survey. Volunteers followed the survey link and were informed of the study's purpose, risks and safeguards, along with information regarding consent, anonymous participation and access to information. The study was described as an exploration of the relationship between positive beliefs and eating‐related thoughts and behaviours. After obtaining consent, participants were invited to complete demographic questions followed by psychometric measures. Contact details of suitable support services were provided within the survey. Following completion of the questionnaire, participants were given the opportunity to enter a monetary prize draw ($50 AUD Amazon voucher). To maintain confidentiality, prize draw was via an external survey link, and the winner was chosen randomly by an individual who was independent from the current study.

### 3.5 Statistical analyses

Results were considered significant at *p* < .05. Statistical Package for the Social Sciences (SPSS; Version 27) program was used for all statistical analyses. To assess whether early adaptive schemas negatively predicted eating disorder symptomatology, four separate hierarchical multiple regression analyses were conducted. To control for their possible influence on the outcome variable, age and BMI were entered in the first block, following previous eating disorder research (e.g., Cooper & Proudfoot, 2013; Dingemans et al., 2006). The second block included the 14 subscales from the YPSQ. The four subscales (Restraint, Eating Concern, Shape Concern and Weight Concern) from the EDE‐Q were entered as the outcome variables in separate hierarchical multiple regression analyses.

## 4 RESULTS

### 4.1 Preliminary analysis

An a priori power analysis was calculated using G*Power (Faul et al., 2007). The sample size for the current study (*n* = 388) was considered adequate given the analysis indicated that 143 would be the required sample to detect a medium effect size (ƒ^2^ = 0.15) using standard alpha (*α* = .05), power of .80 and 16 predictor variables. Prior to conducting the primary analyses, data were screened for univariate and multivariate outliers and violations of normality. The assumptions of linearity, homoscedasticity, collinearity, independence of errors and multivariate normality were met.

Descriptive statistics and correlations between study variables are presented in Table 2. Mean early adaptive schema scores were slightly below normative values (Louis et al., 2018), whereas mean EDE‐Q subscale scores were above normative values (Fairburn & Beglin, 1994). Participants with higher early adaptive schema scores were more likely to be older and report lower levels of eating disorder pathology. Correlations between early adaptive schema and EDE‐Q subscale scores were strong and in the expected negative direction. Early adaptive schema subscales exhibited intercorrelations ranging from .44 to .82 (*p <* .01), while the EDE‐Q subscales exhibited intercorrelations ranging from .69 to .80 (*p <* .01).

**TABLE 2:** Summary of means, standard deviations and bivariate correlations between study variables (*N* = 388)

Variable	*M*	*SD*	1	2	3	4	5	6	7	8	9	10	11	12	13	14	15	16	17	18	19	20
Age	25.7	7.04	‐																			
BMI	19.76	5.59	.32	‐																		
Restraint	3.83	1.96	−.23	−.13	‐																	
Eating concern	3.52	1.75	−.32	‐.08^a^	.72	‐																
Shape concern	4.69	1.85	−.19	.07^a^	.69	.81	‐															
Weight concern	4.44	1.82	−.18	.09^a^	.70	.81	.92	‐														
Emotional fulfilment	3.34	1.08	.25	.08^a^	−.36	−.49	−.50	−.47	‐													
Success	3.66	0.96	.23	.05^a^	−.34	−.47	−.46	−.45	.74	‐												
Empathic consideration	3.58	1.01	.27	.08^a^	−.34	−.44	−.44	−.41	.81	.65	‐											
Optimism	3.55	1.02	.33	.11	−.27	−.43	−.40	−.37	.74	.69	.63	‐										
Emotional openness	3.57	1.08	.27	.11	−.41	−.56	−.54	−.51	.72	.71	.61	.64	‐									
Self‐compassion	3.65	0.98	.22	‐.01^a^	−.33	−.42	−.44	−.44	.60	.60	.60	.55	.67	‐								
Healthy boundaries	3.55	1.13	.25	.02^a^	−.30	−.52	−.49	−.46	.69	.71	.55	.78	.71	.52	‐							
Social belonging	3.44	1.07	.27	.10	−.41	−.53	−.51	−.50	.82	.78	.73	.68	.76	.69	.65	‐						
Healthy self‐control	3.48	0.93	.24	.02^a^	−.33	−.43	−.46	−.44	.76	.66	.77	.68	.64	.67	.61	.69	‐					
Realistic expectations	3.89	1.09	.25	.06^a^	−.28	−.45	−.42	−.36	.72	.65	.65	.73	.67	.47	.70	.64	.70	‐				
Self‐directedness	3.91	1.10	.31	.15	−.29	−.42	−.37	−.33	.71	.62	.67	.79	.60	.55	.64	.63	.70	.75	‐			
Self‐care	3.49	1.31	.18	.05^a^	−.36	−.46	−.50	−.47	.77	.71	.69	.62	.71	.61	.61	.75	.63	.60	.56	‐		
Stable attachment	3.56	1.01	.21	.01^a^	−.29	−.44	−.43	−.42	.73	.69	.67	.68	.61	.49	.65	.71	.68	.66	.62	.61	‐	
Self‐reliance	3.63	1.10	.24	.07^a^	−.32	−.50	−.50	−.46	.68	.73	.58	.67	.75	.56	.70	.71	.57	.64	.60	.67	.59	‐

Abbreviations: BMI, body mass index; *M*, mean; *SD*, standard deviation.

^a^ *p* > .05. All other correlations *p* < .001.

### 4.2 Primary analyses

Four separate hierarchical multiple regression analyses were conducted to assess which, if any, early adaptive schemas predicted eating disorder symptomatology, as measured by the four EDE‐Q subscales.

#### 4.2.1 Restraint

In predicting Restraint, as shown in Table 3, the first block included age and BMI, which accounted for significant variability in Restraint, *R* ^*2*^ = .04, *F*(1, 383) = 15.61, *p* < .001. The addition of the 14 early adaptive schema subscales in the second block significantly accounted for an additional 16% of variance in Restraint scores, ∆*R* ^*2*^ = .16, ∆*F*(14, 369) = 5.25, *p* < .001. The overall model significantly predicted Restraint, *R* ^*2*^ = .20, *F*(15, 369) = 6.10, *p* < .001. By Cohen's (Cohen, 1988) conventions, a combined effect of this magnitude is medium (ƒ^2^ = .26). Of the 14 subscales, Optimism was the strongest predictor, accounting for 1.3% of significant unique variance in Restraint scores. That is, increased Optimism was associated with more Restraint. All other subscales did not contribute significantly to the variance in Restraint scores.

**TABLE 3:** Hierarchical multiple regression analysis predicting restraint from early adaptive schemas

Variable	*B* [95% CI]	*SE B*	*β*	*R* ^2^	*∆R* ^2^
Step 1				.04	.04
Constant	8.65 [5.47, 11.84]	1.62			
Age	−0.05 [−0.08, −0.02]	0.01	−.19*		
BMI	−0.02 [−0.06, 0.02]	0.02	−.05		
Step 2				.20	.16
Constant	12.81 [8.35, 17.27]	2.27			
Age	−0.03 [−0.05, 0.00]	0.01	−.09*		
BMI	−0.02 [−0.06, 0.01]	0.02	−.06		
Emotional fulfilment	−0.11 [−0.52, 0.30]	0.21	−.06		
Success	−0.02 [−0.38, 0.34]	0.19	−.01		
Empathic consideration	−0.02 [−0.37, 0.33]	0.18	−.01		
Optimism	0.39 [0.04, 0.75]	0.20	.19*		
Emotional openness	−0.36 [−0.69, −0.03]	0.18	−.20		
Self‐compassion	−0.02 [−0.32, 0.28]	0.16	−.01		
Healthy boundaries	−0.28 [−0.58, −0.02]	0.16	−.16		
Social belonging	−0.33 [−0.70, 0.05]	0.19	−.18		
Healthy self‐control	−0.09 [−0.46, 0.29]	0.20	−.04		
Realistic expectations	−0.09 [−0.23, 0.41]	0.17	.05		
Self‐directedness	−0.14 [−0.45, 0.17]	0.16	−.08		
Self‐care	−0.10 [−0.34, 0.14]	0.12	−.07		
Stable attachment	−0.05 [−0.26, 0.35]	0.16	.02		
Self‐reliance	0.17 [−0.12, 0.46]	0.15	.10		

Abbreviations: *β*, standardized beta coefficient; *B*, unstandardized beta coefficient; BMI, body mass index; CI, confidence interval; *SE*, standard error.

* *p* < .05.

#### 4.2.2 Eating concern

In predicting Eating Concern, as shown in Table 4, the first block included age and BMI, which significantly accounted for variability in Eating Concern, *R* ^*2*^ = .10, *F*(1, 383) = 41.95, *p* < .001. The addition of the 14 early adaptive schema subscales significantly accounted for an additional 27% of variance in Eating Concern, ∆*R* ^*2*^ = .27, ∆*F*(14, 369) = 11.04, *p* < .001. The overall model significantly predicted Eating Concern, *R* ^*2*^ = .37, *F*(15, 369) = 14.12, *p* < .001, Cohen's *f* ^2^ = .57. Of the 14 subscales, Optimism, Emotional Openness and Spontaneity, Social Belonging and Healthy Boundaries were the strongest predictors, accounting for 1.2%, 1%, 1% and 2.3% of significant unique variance in Eating Concern scores, respectively. That is, increased Emotional Openness and Spontaneity and Healthy Boundaries were associated with less Eating Concern, while higher Optimism was associated with greater Eating Concern. All other subscales did not contribute significantly to the variance in Eating Concern scores.

**TABLE 4:** Hierarchical multiple regression analysis predicting eating concern from early adaptive schemas

Variable	*B* [95% CI]	*SE B*	*β*	*R* ^2^	*∆R* ^2^
Step 1				.10	.10
Constant	5.64 [5.01, 6.27]	0.32			
Age	−0.08 [−0.10, −0.06]	0.01	−.31		
BMI	0.02 [−0.01, 0.06]	0.01	.06		
Step 2				.37	.27
Constant	7.82 [7.04, 9.00]	0.40			
Age	−0.04 [−0.07, −0.02]	0.01	−.17		
BMI	0.02 [−0.01, 0.05]	0.01	.05		
Emotional fulfilment	−0.03 [−0.35, 0.30]	0.17	−.02		
Success	−0.09 [−0.19, 0.37]	0.15	.05		
Empathic consideration	−0.03 [−0.31, 0.24]	0.14	−.02		
Optimism	0.39 [0.10, 0.68]	0.15	.23*		
Emotional openness	−0.29 [−0.55, −0.02]	0.14	−.18*		
Self‐compassion	0.00 [−0.23, 0.23]	0.12	.001		
Healthy boundaries	−0.43 [−0.67, −0.20]	0.12	−.28***		
Social belonging	−0.27 [−0.56, 0.00]	0.15	−.16*		
Healthy self‐control	−0.05 [−0.35, 0.24]	0.16	−.03		
Realistic expectations	−0.11 [−0.36, 0.14]	0.13	−.07		
Self‐directedness	−0.02 [−0.26, 0.23]	0.13	−.01		
Self‐care	−0.06 [−0.24, 0.13]	0.10	−.04		
Stable attachment	−0.04 [−0.28, 0.20]	0.13	−.02		
Self‐reliance	−0.05 [−0.28, 0.18]	0.12	−.03		

Abbreviations: *β*, standardized beta coefficient; *B*, unstandardized beta coefficient; BMI, body mass index; CI, confidence interval; *SE*, standard error.

* *p* < .05.

** *p* < .01.

*** *p* < .001.

#### 4.2.3 Shape concern

In predicting Shape Concern, as shown in Table 5, the first block included age and BMI, which significantly accounted for variability in Eating Concern, *R* ^*2*^ = .03, *F*(1, 383) = 12.89, *p* < .001. The addition of the 14 early adaptive schema subscales significantly accounted for an additional 31% of variance in Shape Concern, ∆*R* ^*2*^ = .31, ∆*F*(14, 369) = 12.36, *p* < .001. The overall model significantly predicted Shape Concern, *R* ^*2*^ = .34, *F*(15, 369) = 12.75, *p* < .001, Cohen's ƒ^2^ = .52. Of the 14 subscales, Optimism, Healthy Boundaries and Self‐Care were the strongest predictors, significantly accounting for 1.2%, 1.4% and 1% of unique variance in Shape Concern scores, respectively. That is, greater Healthy Boundaries and Self‐Care scores were associated with less Shape Concern, while higher Optimism was associated with greater Shape Concern. All other subscales did not contribute significantly to the variance in Shape Concern scores.

**TABLE 5:** Hierarchical multiple regression analysis predicting shape concern from early adaptive schemas

Variable	*B* [95% CI]	*SE B*	*β*	*R* ^2^	*∆R* ^2^
Step 1				.03	.03
Constant	6.01 [5.32, 6.69]	0.35			
Age	−0.05 [−0.07, −0.02]	0.01	−.18**		
BMI	0.05 [0.02, 0.09]	0.02	.15**		
Step 2				.34	.31
Constant	8.57 [7.74, 9.40]	0.42			
Age	−0.01 [−0.04, 0.01]	0.01	−.05		
BMI	0.04 [0.01, 0.08]	0.01	.04**		
Emotional fulfilment	−0.25 [−0.59, 0.10]	0.18	−.15		
Success	−0.12 [−0.19, 0.41]	0.16	.06		
Empathic consideration	−0.04 [−0.26, 0.33]	0.15	.02		
Optimism	0.41 [0.10, 0.72]	0.16	.23*		
Emotional openness	−0.19 [−0.47, 0.09]	0.15	−.12		
Self‐compassion	−0.10 [−0.34, 0.15]	0.13	−.05		
Healthy boundaries	−0.36 [−0.61, −0.11]	0.13	−.23**		
Social belonging	−0.05 [−0.36, 0.27]	0.16	−.03		
Healthy self‐control	−0.22 [−0.53, 0.09]	0.17	−.11		
Realistic expectations	0.02 [−0.25, 0.29]	0.14	.01		
Self‐directedness	−0.04 [−0.22, 0.30]	0.13	.03		
Self‐care	−0.20 [−0.40, 0.0]	0.10	−.14*		
Stable attachment	−0.07 [−0.33, 0.18]	0.13	−.04		
Self‐reliance	−0.19 [−0.43, 0.05]	0.13	−.11		

Abbreviations: *β*, standardized beta coefficient; *B*, unstandardized beta coefficient; BMI, body mass index; CI, confidence interval; *SE*, standard error.

* *p* < .05.

** *p* < .01.

#### 4.2.4 Weight concern

In predicting Weight Concern, as shown in Table 6, the first block included age and BMI, which significantly accounted for variability in Weight Concern, *R* ^*2*^ = .03, *F*(1, 383) = 12.24, *p* = .001. The addition of the 14 early adaptive schema subscales significantly accounted for an additional 28% of variance in Weight Concern, ∆*R* ^*2*^ = .28, ∆*F*(14, 369) = 10.68, *p* < .001. The overall model significantly predicted Weight Concern, *R* ^*2*^ = .31, *F*(15, 369) = 11.07, *p* < .001, Cohen's ƒ^2^ = .45. Of the 14 subscales, Optimism and Healthy Boundaries were the strongest predictors, significantly accounting for 1.1% and 1.5% of unique variance in Weight Concern scores, respectively. That is, increased Healthy Boundaries was associated with less Weight Concern, while higher Optimism was associated with greater Weight Concern. All other subscales did not contribute significantly to the variance in Weight Concern scores.

**TABLE 6:** Hierarchical multiple regression analysis predicting weight concern from early adaptive schemas

Variable	*B* [95% CI]	*SE B*	*β*	*R* ^2^	*∆R* ^2^
Step 1				.03	.03
Constant	5.72 [5.03, 6.40]	0.35			
Age	−0.05 [−0.07, −0.02]	0.01	−.18**		
BMI	0.06 [0.03, 0.10]	0.02	.18**		
Step 2				.31	.28
Constant	8.15 [7.30, 8.99]	0.43			
Age	−0.02 [−0.04, 0.01]	0.01	−.06*		
BMI	0.05 [0.02, 0.09]	0.01	.15**		
Emotional fulfilment	−0.17 [−0.52, 0.18]	0.18	−.10		
Success	0.06 [−0.25, 0.37]	0.16	.03		
Empathic consideration	0.04 [−0.26, 0.34]	0.15	.02		
Optimism	0.40 [0.08, 0.72]	0.17	.22*		
Emotional openness	−0.19 [−0.48, 0.10]	0.15	−.11		
Self‐compassion	−0.14 [−0.40, 0.11]	0.13	−.08		
Healthy boundaries	−0.37 [−0.63, −0.11]	0.13	−.23**		
Social belonging	−0.11 [−0.43, 0.21]	0.17	−.06		
Healthy self‐control	−0.27 [−0.59, 0.08]	0.17	−.14		
Realistic expectations	0.14 [−0.13, 0.41]	0.14	.09		
Self‐directedness	−0.14 [−0.13, 0.41]	0.14	.08		
Self‐care	−0.15 [−0.35, 0.06]	0.10	−.11		
Stable attachment	−0.16 [−0.42, −0.06]	0.14	−.09		
Self‐reliance	−0.16 [−0.41, 0.08]	0.13	.10		

Abbreviations: *β*, standardized beta coefficient; *B*, unstandardized beta coefficient; BMI, body mass index; CI, confidence interval; *SE*, standard error.

* *p* < .05.

** *p* < .01.

## 5 DISCUSSION

To our knowledge, this is the first study to investigate the relationship between early adaptive schemas and eating disorder symptomatology in adults and provides a preliminary contribution to the understanding of how positive beliefs may be protective against eating disorder cognitions and behaviours. The findings indicated that five of the 14 early adaptive schemas predicted eating disorder outcome measures. In support of the exploratory hypothesis, higher levels of Healthy Boundaries, Emotional Openness and Spontaneity, Social Belonging and Self‐Care predicted lower levels of eating disorder symptoms across various EDE‐Q subscales. Unexpectedly, higher levels of Optimism were associated with worse eating disorder pathology across all subscales. Overall, the findings provide evidence for certain early adaptive schemas as novel concepts that may mitigate the development of cognitions and behaviours associated with eating disorder symptomatology.

Given the paucity of research examining early adaptive schemas and markers of eating disorder pathology, the findings may be viewed through the lens of positive clinical psychology and schema theory. In general, the findings partially align with the body of schema literature in the eating disorder research field. While the present study identified five of the 14 early adaptive schemas as significant predictors of various eating disorder outcomes, previous research has consistently reported strong positive associations between the majority of early maladaptive schemas, except Entitlement and various eating disorder measures (e.g., De Paoli et al., 2017; Legenbauer et al., 2018; Leung & Price, 2007; Maher et al., 2022; Sines et al., 2008). That is, individuals who develop negative core beliefs in early life endorse more severe eating disorder cognitions and behaviours. This discrepancy between the present findings and previous research may be explained by preliminary early adaptive schemas research by Louis and colleagues, who suggest that adaptive schemas and maladaptive schemas are separate constructs and can co‐exist (Louis et al., 2018). That is, individuals can endorse both adaptive and maladaptive schemas, despite being theoretical counterparts. This notion aligns with Louis et al.'s (2018) assertion that adaptive and maladaptive schemas are activated by different experiences, both health and unhealthy, and do not cluster together within the cognitive schema network (Louis et al., 2018). Such findings suggest that both adaptive and maladaptive schemas can exist simultaneously and are not merely polar opposites of one another. This assertion was examined in a recent study, which found that all early adaptive schemas provided predictive utility of mental health outcomes above and beyond that of early maladaptive schemas (Louis et al., 2018). Within the eating disorder context, the findings suggest that a unique set of early adaptive schemas, developed via positive early life experiences (e.g., secure attachment relationships, validation of emotional expression, autonomy), may mitigate the development of eating disorder cognitions and behaviours, even in the presence of unmet emotional needs.

Examination of the findings from a schema theory lens may further explain the negative relationship between five of the early adaptive schemas and eating disorder outcome measures. Young et al. (2003) proposed that adverse childhood experiences involving poor caregiver attachment and bonding experiences lead to the development of unconditional beliefs that shape an individual's perspective of themselves and the world. This aetiological mechanism of psychopathology has been explored in eating disorder research. For example, individuals with an eating disorder diagnosis were more likely to report having an insecure attachment style (De Paoli et al., 2017; Illing et al., 2010; Tasca et al., 2013) and perceived their parents to be less caring and overly controlling (Brown et al., 2016; Deas et al., 2011; Leung et al., 2000). Taken together, such findings suggest that individuals endorsing worse eating disorder symptomatology likely experienced less positive interactions with attachment figures, which hindered the development of unconditional positive core beliefs.

This notion is further reinforced by attachment theory, which suggests that repeated interactions with attachment figures lead to internal working models in children and become the basis of affect regulation and interpersonal behavioural styles in adult life (Bowlby, 1978). These findings align with the present study, which found that lower levels of Emotional Openness and Social Belonging predicted higher levels of Eating Concern. Indeed, the transdiagnostic model purports that poor affect regulation and interpersonal relationships, among other components, are key maintaining factors of eating disorder pathology (Fairburn et al., 2003). Research has shown that individuals who endorsed positive social beliefs (e.g., I fit into a group; I am a friendly person) reported fewer eating disorder symptoms (Cooper & Proudfoot, 2013). In contrast, clinical eating disorder samples have consistently reported significantly fewer social group memberships, higher levels of rejection sensitivity, increased social isolation, fear of being ostracized and worse interpersonal functioning than their nonclinical counterparts (e.g., Arcelus et al., 2011; De Paoli et al., 2017; Keith et al., 2009; Meneguzzo et al., 2020; Rowlands et al., 2021). Moreover, eating disorder symptomatology has been associated with poor distress tolerance, emotional instability and difficulty experiencing both pleasant and unpleasant emotions (e.g., Hambrook et al., 2011; Hovrud et al., 2020; Overton et al., 2005).

Building on this premise, Waller and colleagues proposed that eating disorder behaviours are used to avoid negative affect associated with schema activation and attachment related distress (Waller et al., 2007). Although the model focused heavily on schemas processes, the model's assertions align with research that shows that eating disorder samples display higher levels of avoidance coping (e.g., Vanzhula et al., 2020). Taken together, it appears that individuals who experience positive early childhood experiences may go on to develop healthy affect regulation and interpersonal skills, which reduces their need to use dysfunctional eating disorder behaviours to avoid distressing affect.

An alternative perspective suggests that metacognitive theory may help to explain the relationship between eating disorder symptomatology and core beliefs. In a recent literature review, Mansueto and colleagues sought to understand the development of metacognitions in childhood and adolescence. A total of five studies reported that exposure to childhood adversities such as emotional abuse, emotional neglect or physical abuse was associated with the development of both positive and negative metacognitions in childhood and adolescence (Mansueto et al., 2019). The authors posited that pervasive exposure to threat and danger resulted in coping strategies such as hypervigilance, emotional dysregulation and perceived lack of internal control. Such findings can be related to the current study such that individuals with higher eating disorder scores may be more likely to develop early maladaptive schemas, and similarly, positive and negative metacognitive beliefs, due to their potential exposure to childhood adversity.

Interestingly, high levels of Optimism consistently predicted higher levels of eating disorder psychopathology across all EDE‐Q subscales. That is, less worrying and perceived lack of vulnerability (i.e., Optimism) were associated with worse eating disorder psychopathology, which is not supported by previous research. For example, a recent study found that optimistic individuals reported lower levels of cognitive restraint and emotional eating and had a lower risk of developing an eating disorder (Robert et al., 2020). Additionally, Foye and colleagues reported that individuals with higher levels of optimism were less likely to endorse disordered eating attitudes (Foye et al., 2019). This aligns with research examining the theoretical early maladaptive schema counterpart of Optimism (i.e., Vulnerability to Harm and Pessimism), which found that individuals with an eating disorder diagnosis reported significantly higher Pessimism and Vulnerability scores than their nonclinical counterparts (Elmquist et al., 2015). That is, they were more likely to report higher levels of negativity, hopelessness and fear about their future. A possible explanation for the discrepancy in findings is that activation of certain early adaptive schemas, in combination with pre‐existing early maladaptive schemas, may present as a specific overcompensatory eating disorder coping style that is conceptualized as excessive optimism. This coping style functions to maintain eating disorder symptomatology through denial of various aspects of the eating disorder and avoidance of emotional expression that may lead to rejection or criticism (Simpson & Smith, 2020). This assertion aligns with the present study, as lower levels of Emotional Openness were associated with higher levels of Eating Concern. The finding should also be interpreted with caution, given that Optimism only accounted for 1% of variance in eating disorder psychopathology.

### 5.1 Limitations and future research

Several limitations need to be considered in the present study. First, the correlational study design indicates that causation and direction of relationships cannot be inferred. Although schema theory proposes that schemas are formed in early childhood and adolescence, it may be the case that the development of weight and shape‐related beliefs occurred prior to the development of early adaptive schemas. This is especially relevant, given that children are being exposed to body‐related attitudes via parents and media at younger ages (Daragnova, 2013). Future research should employ longitudinal study designs to elucidate the nature and direction of the relationship between early adaptive schemas and eating disorder symptomatology.

Due to the exploratory nature of the present study, differences in early adaptive schemas between eating disorders diagnostic groups were not examined. Given the differences in schema processes between diagnostic groups (e.g., Waller et al., 2007), future research is required to elucidate whether differences also exist for early adaptive schemas. Such research may inform future treatment choices according to eating disorder presentation. Moreover, given the samples high proportion of participants reporting a current eating disorder diagnosis, this study was unable to examine difference between clinical and nonclinical groups. Given that schema research has consistently reported significant differences between such groups when examining disorders such as depression, future research is required to understand potential differences, especially in developing target outcomes for eating disorder treatment. Furthermore, the sample was made up of 90% females. Given the previously documented differences between gendered eating disorder cognitions, the findings from the present study cannot be extrapolated to the male population, highlighting an avenue for future research.

Finally, it should be highlighted that although a combined predictive variance of between 16% and 31% was observed for all subscales, each early adaptive schemas accounted for less than 2% of the variance in eating disorder symptomatology scores. Little is known about the nature of the relationship between positive psychological constructs and eating disorder outcomes. Despite a large body of schema therapy research validating the presence of early maladaptive schemas in individuals with eating disorders, the present findings concerning the predictive nature of specific early adaptive schemas on eating disorder outcomes remain putative due to the complexity of factors that contribute to eating disorder aetiology and maintenance. For example, while disrupted attachment relationships and unmet emotional needs have been consistently implicated in eating disorder pathology, research has identified factors such as genetics and trait perfectionism as potential covariates. Moreover, the present findings should not be exclusively interpreted in the context of schema therapy, especially given Louis et al. (2018) suggestions about the proposed mechanism of action for early adaptive schemas in predicting mental health outcomes. For example, the researchers suggest that while early adaptive schemas may be theoretical counterparts of their opposite early maladaptive schemas, they also propose the notion that early adaptive schemas may be unique constructs that develop in childhood and adolescence and cluster separately to early maladaptive schemas in the brain. Future research should focus on validating the YSPQ in clinical eating disorder samples. Given the observed differences in early maladaptive schema scores between eating disorder and dieting samples despite reported similarities in weight and shape concern (e.g., Cooper & Turner, 2000; Leung & Price, 2007), it is evident that schema content is unique in eating disorders. Therefore, future studies are required to clarify the nature of early adaptive schemas in eating disorder samples.

### 5.2 Clinical implications

Within the burgeoning field of positive psychology, the concept of early adaptive schemas offers a complementary conceptualization to the existing knowledge of eating disorder aetiology and treatment. Early maladaptive schemas provide insight into unmet core emotional needs in early life that may contribute to the development of unhealthy shape and weight‐related beliefs. In contrast, early adaptive schemas shed light on significant positive childhood and adolescent experiences that may contribute to the mitigation of eating disorder symptomatology and possibly development of positive beliefs about one's body and shape. This study provides preliminary evidence of specific early adaptive schemas associated with lower levels of eating disorder symptomatology. Within the context of the treatment setting, early adaptive schema measures may function as a complementary therapeutic target, alongside early maladaptive schemas. Moreover, focussing on early positive experiences that have contributed to adaptive unconditional core beliefs may improve weight‐ and shape‐related beliefs about oneself in the treatment of eating disorders.

## 6 CONCLUSION

The present study found that greater Healthy Boundaries were associated with lower levels of eating disorder psychopathology. In contrast, greater Optimism was associated with worse eating disorder psychopathology. Moreover, Emotional Openness and Social Belonging and Self‐Care were important in predicting lower levels Eating Concern and Shape Concern, respectively. Given that this is the first study to demonstrate the relationship between early adaptive schemas and eating disorder cognitions and behaviours, this evidence, although putative, sets the stage for future research to identify causal effects of early adaptive schemas on eating disorder symptomatology.

## CONFLICT OF INTEREST

The authors wish to declare that no conflict of interest exists.

## Data Availability

The data that support the findings of this study are available from the corresponding author, Andrew Allen, upon reasonable request.
